# Stress distribution of different lumbar posterior pedicle screw insertion techniques: a combination study of finite element analysis and biomechanical test

**DOI:** 10.1038/s41598-021-90686-6

**Published:** 2021-06-21

**Authors:** Mingzhi Song, Kebin Sun, Zhonghai Li, Junwei Zong, Xiliang Tian, Kai Ma, Shouyu Wang

**Affiliations:** 1 Department of Orthopaedics, The First Affiliated Hospital of Dalian Medical University, No.222 Zhongshan Road, Xigang District, Dalian, Liaoning China; 2 Department of Orthopaedics, The Third Affiliated Hospital of Dalian Medical University, No.378 West Section Shiji Road, Pulandian District, Dalian, Liaoning China; 3 College of Information Engineering, Dalian University, No.10 Xuefu Avenue, Economic & Technical Development Zone, Dalian, Liaoning China

**Keywords:** Computer science, Experimental models of disease

## Abstract

At present, the pedicle screw is the most commonly used internal fixation device. However, there are many kinds of common posterior pedicle screw insertion techniques performed to reconstruct the lumbar stability. Therefore, spinal surgeons often face a difficult choice. The stress distribution of internal fixation system is an important index for evaluating safety. Unfortunately, little had been known about the difference of stress distribution of screw-rod systems that established by Roy-Camille, Magerl and Krag insertion techniques. Here, combination of finite element analysis and model measurement research was adopted to evaluate the difference of stress. Following different pedicle screw insertion techniques, three lumbar posterior surgery models were established after modeling and validation of the L1–S1 vertebrae finite element model. By analyzing the data, we found that stress concentration phenomenon was in all the postoperative models. Roy-Camille and Magerl insertion techniques led to the great stress on screw-rod systems. Then, fresh frozen calf spines were selected as a model for subsequent measurements. Fitted with a specially designed test pedicle screw, L5–L6 vertebrae were selected to repeat and verify the results of the finite element analysis. With the aid of universal testing machine and digital torque wrench, models simulated flexion, extension, lateral bending and rotation. Finally, the strain value was captured by the strain gauge and was then calculated as the stress value. Krag and Magerl were found to be the safer choice for pedicle screw insertion. Overall, our combination method obtained the reliable result that Krag insertion technique was the safer approach for pedicle screw implantation due to its relatively dispersive stress. Therefore, without the consideration of screw size, pedicle fill, bone density, and bone structures, we recommend the Krag insertion technique as the first choice to reconstruction of lumbar stability. Additionally, the combination method of finite element analysis and strain gauge measurement can provide a feasible way to study the stress distribution of spinal internal fixation.

## Introduction

Lower back pain, the second common ailment, happens to almost 80% people all over the world^1^. Trauma, Congenital disease, tumor, infections and other degenerative reason may cause lower back pain. Due to a fracture, the lumbar spine may become unstable. And operations on most of the diseases often destroy the stability of lumbar spine. Therefore, reconstrucion of lumbar stability is the key to treatment. Due to the great loading conditions, lumbar region reconstruction is usually accomplished with the posterior pedicle screw-rod system.

In 1959, Boucher first described the pedicle screw instrumentations^2^. After a long period of development and improvement, they have been widely used for providing immediate stability, correcting deformity, enhancing bony fusion and maintaining normal spinal segments. Based on this, the pedicle fixation technique was able to treat deformations, tumors, unstable fractures, tuberculosis and degenerative disorders of the spine. With the increasement of clinical applications of pedicle screws, postoperative problems including breakage, loosening, improper placement, spinal cord injury, nerve root injury, dural tears, pseudarthrosis and instrumentation infection gradually appear^3–7^. The most common postoperative problem is breakage, which resulted from screw fracture due to torsion or bending. The previous studies show that screw breakage often occurs around the thread-shank region and has an incidence of 2.6% to 60%^8–10^. In recent years, the safety of pedicle screw and connecting rods have been improved by application of new shape, hard material and motorization, but case reports of breakage are not rare^11,12^. Protecting pedicle screws and connecting rod from breakage is still needed to be resolved.

**Figure 1:**
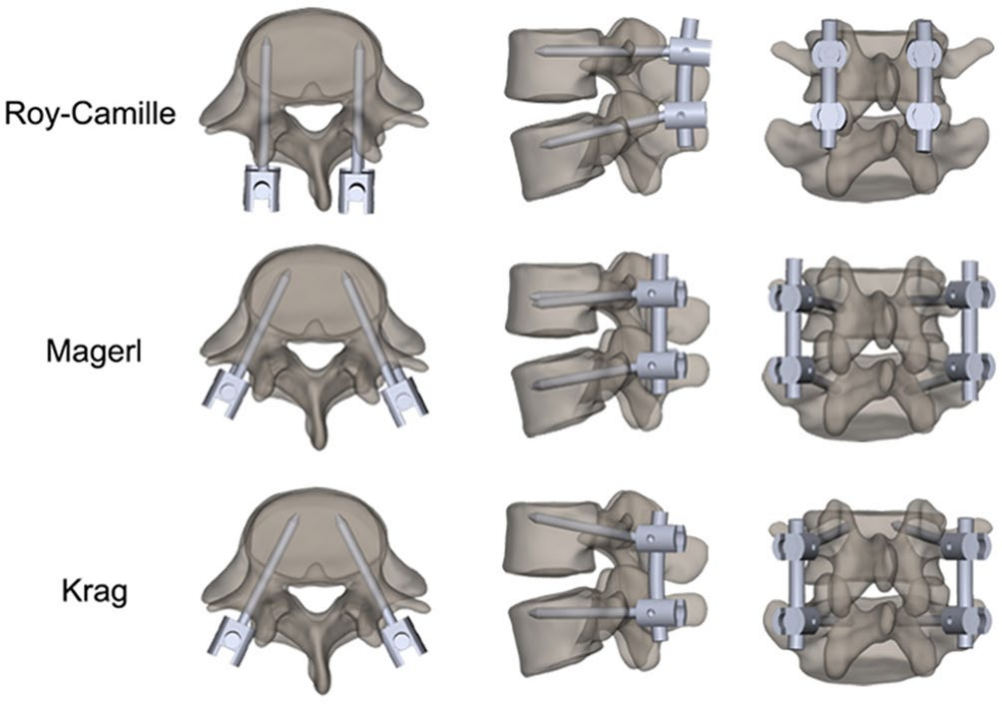
The methods of three pedicle screw insertion techniques. From top to bottom, they are Roy-Camille, Magerl, and Krag, respectively.

Researchers experimenting on biomechanical testing of lumbar spine notice that the breakage is relevant to the difference of various internal fixation systems^13^. Furthermore, the retrospective study has achieved the same result^14^. Although researchers have shown that screw size, pedicle fill, bone density, bone structures, and insertion technique are important factors for influencing internal fixation stability^15–17^. Unfortunately, there is still no adequate evaluation on the biomechanical features of different pedicle screw insertion techniques. Under circumstance of using the same pedicle screw, the choice of the insertion technique varies with different surgeons. They prefer for paying more attention for intraoperative safety during selecting insertion technique. But the resulting breakage risk of internal fixation system will be unconsciously neglected. In fact, insertion technique has three key elements including position, orientation and depth. As mentioned in the previous literature, different techniques with different elements will have effect on the stress state of inserted screw instrumentations^18^. Therefore, from the perspective of biomechanics, performing the low stress technique may reduce the breakage risk as well as ensure the success of the operation^19^. With simulation, veracity and repeatability, the finite element (FE) analysis has been viewed as a reliable approach for evaluating the biomechanical behavior of different internal fixation system. More intuitive data can be obtained from traditional cadaver research, which is also very important for biomechanical study. In the following research, three pedicle screw insertion techniques will selected as the research objects. Roy-Camille, Magerl, and Krag are three common and typical insertion techniques that are performed for stabilizing the lumbar spine^20–22^ (Fig. 1). Each of these three techniques has respective characteristics of entry point and insertion orientations. Here, we carry out a FE analysis and a series of model measurement researches to unveil the biomechanical difference among different insertion techniques. The primary objectives of this study were to (1) establish a feasible FE model of the lumbar spine; (2) simulate three pedicle screw insertion techniques on the FE model; (3) compare the stress distribution of the posterior screw-rod system following different insertion techniques; (4) establish measured models with different posterior screw-rod system; (5) simulate six various working conditions and collect the maximum strain values on the internal fixations of each model; (6) comprehensively analyze the results of both FE analysis and biomechanical tests to identify the safe insertion technique.

## Materials and methods

### Intact finite element model

**Figure 2:**
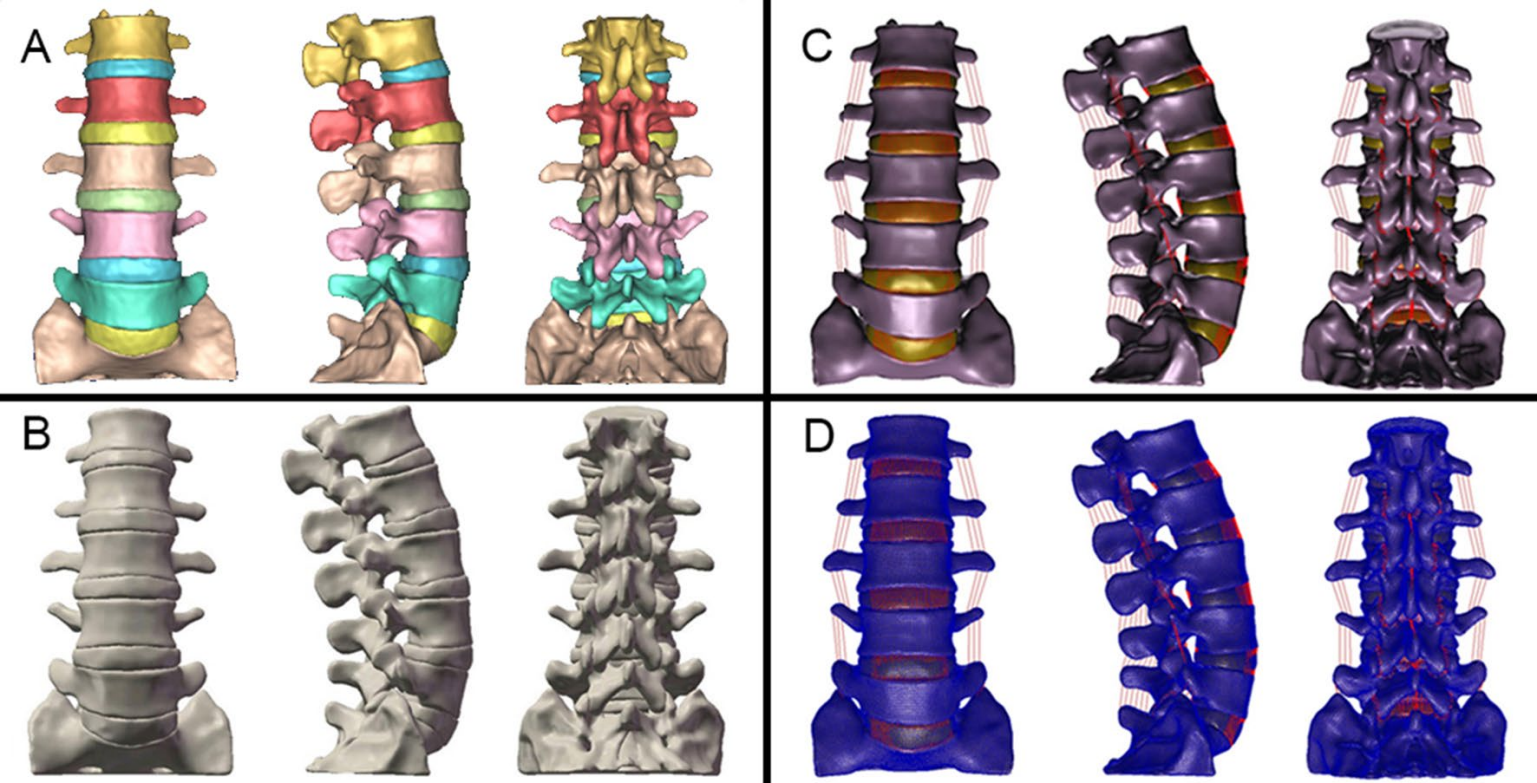
The procedure to establish the intact FE model of L1–S1 vertebrae. (**A**) a rough 3D model of L1–S1 vertebrae, (**B**) the rational non-uniform geometry structure of the model, (**C** and **D**) setting of the nonlinear simulation of ligaments and the meshing of the model.

This study was assessed and approved by the Ethics Committee of the First Affiliated Hospital of Dalian Medical University (approval number: YJ-FB-2016-45). The study was carried out in accordance with the relevant guidelines and regulations, and an informed consent was obtained from the subject. Geometrical details were obtained from a thirty-year-old healthy male volunteer in an unloaded neutral position by high-resolution computed tomography. Including five lumbar vertebrae, parts of sacral vertebrae and five intervertebral disks, the Dicom data, was imported into the Mimics 10.01 software (Materialise Inc., Leuven, Belgium). A threshold was set to differentiate bone and soft tissue. Boolean calculation and interactive three-dimensional manual/automatic cutting operations were performed to establish a rough three-dimensional (3D) model of L1–S1 vertebrae (Fig. 2A). For both of smoothening uneven surfaces and forming entity, the model was polished, filled, denoised and solidified in Geomagic Studio 12.0 software (Raindrop Geomagic Inc., Morrisville, NC, USA). Then, the rational non-uniform geometry structure was achieved and assembled to the intact model in Solidworks 2012 software (SolidWorks Corp., Waltham, MA, USA) (Fig. 2B). It was imported into Hypermesh 13.0 software (Altair Engineering Inc., Troy, MI, USA) to generate FE meshes (Fig. 2C, D). Patran/Nastran 2012 software (MSC Software Corp., Newport Beach, CA, USA) was performed to define the material properties, set the boundary and loading conditions, calculate conditions and accomplish FE analysis.

Each lumbar vertebra consisted of posterior bone elements, cortical bone elements and cancellous elements. And each intervertebral disc was modeled as a central nucleus surrounded by the annulus fibrosus. The cartilaginous endplate elements were also truly simulated the connection between vertebra and intervertebral disc. Six major lumbar spine ligaments were incorporated into the model: anterior longitudinal ligament, posterior longitudinal ligament, ligamentum flavum, interspinous ligament, capsular ligaments and intertransverse ligament. The ligaments were modeled as nonlinear tension-only connectors via a hypoelastic material designation. The facet articulations of the ten pairs of zygapophyseal joints in L1–S1 vertebrae were modeled as frictionless contact elements, due to its infinitesimal friction.

To improve the simulation quality, the tetrahedral mesh was generated for all the vertebrae and disc models. The material properties of the various tissues used in this FE model were derived from literature^23,24^ and are listed as Table 1.

**Table 1:** Material properties of L1–S1 vertebrae FE model components.

Part	Young’s modulus (MPa)	Poisson’s ratio
Cortical bone	12,000.00	0.30
Cancellous bone	100.00	0.20
Posterior bone	3,500.00	0.25
Annulus	4.20	0.45
Nucleus pulposus	1.00	0.50
Anterior ligament	20.00	0.40
Posterior ligament	20.00	0.40
Interspinous ligament	10.00	0.30
Supraspinous ligament	10.00	0.30
Ligamentum flavum	10.00	0.30
Capsular ligament	10.00	0.30
Transverse ligament	10.00	0.30
Titanium screw and rod	113,000.00	0.30

The boundary conditions applied were as followed: movements at the bottom of the sacrum were constraint, and a series of unified movements was considered. A compressive preload of 500.0 N combined with a pure moment of 10.0 Nm^25^ was applied at the central node on the top side of the first lumbar vertebra to simulate flexion, extension, left–right lateral bending and left–right axial rotation movements.

### Model validation

For most FE analysis studies, simulation results of the intact FE model were compared with the data reported in the previous literatures. Here, simulation results in this study were compared with both existing well-validated FE models reported in the literature and the data of biomechanical test. Generally, it is accepted that greater number and diversity of comparisons between a model and experimental data increases the reliability of validation^26^. For better simulation, this new FE model was tested in loading conditions consisting of both moment and compression in all the six degrees of freedom (flexion, extension, left and right lateral bending, and left and right axial rotations). Range of motion (ROM) was the only parameter chosen for validation. Model validation was accomplished by comparing with in vitro biomechanical test data^27,28^ and simulation results obtained from four well-validated FE models in the literature^25,29–31^.

### Three finite element models of posterior fixation of L4–L5 vertebrae

In Geomagic Studio 12.0 software, simulation models including simplified pedicle screws (diameter = 6.5 mm, length = 45 mm) and connecting rods (diameter = 5.5 mm, length = 35 mm) were designed respectively. Because of the higher incidence of spine diseases, L3–S1 vertebrae of the intact FE model were adopted to simulate the posterior lumbar surgery. With the combination of bilateral connecting rods, four pedicle screws were inserted into the pedicle of vertebral arches according to different methods (Roy-Camille, Magerl, and Krag). Therefore, three different postoperative models of L4–L5 vertebrae could be achieved.

**Figure 3:**
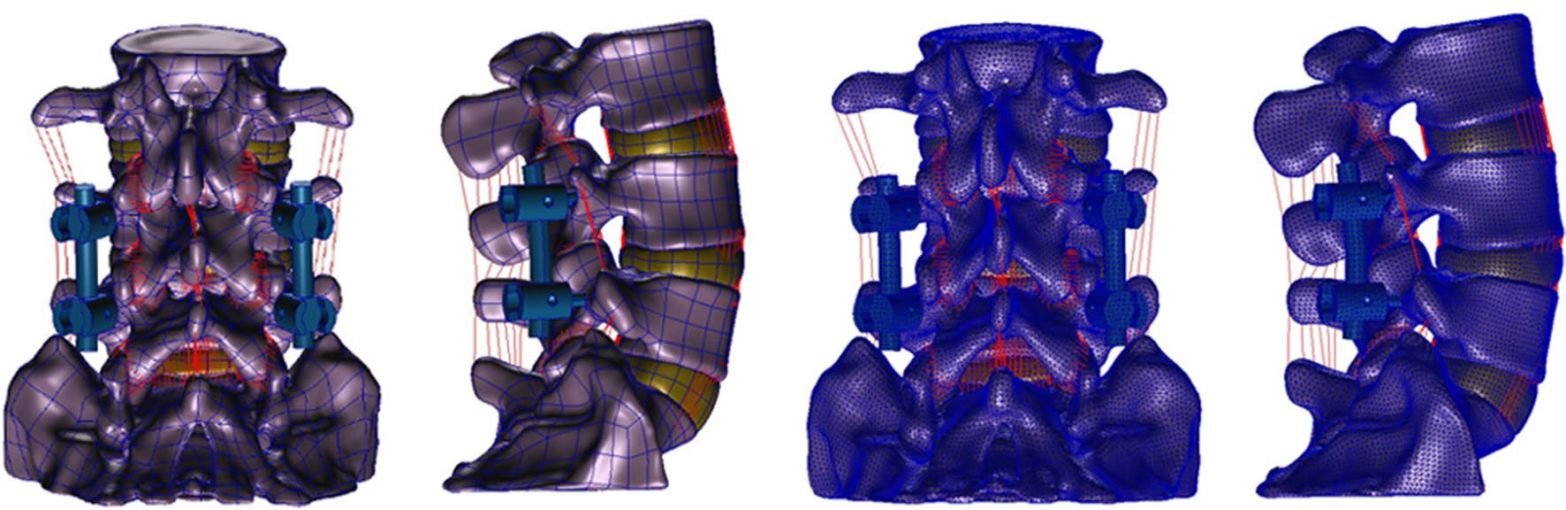
The procedure of lumbar posterior surgery simulation and establishment of the postoperative FE model of L3–S1 vertebrae.

The same as the simulation method of intact FE model, postoperative models also underwent a series procedure of remeshing, defining material properties, and setting boundary and loading conditions (Fig. 3). Considering the safety of postoperative functional exercise, 300 N was selected as the load. Finally, under the vertical compressive preload of 300.0 N on L3 vertebra and a pure moment of 10.0 Nm, six different working conditions (flexion, extension, left and right lateral bending, left and right axial rotations) of all the models were simulated to calculate the stress distribution and intervertebral ROM.

### Biomechanical test model preparation

Nine calf spines (L4–S2) were purchased from the butcher. Radiological examination was performed to exclude spinal disease for these spines. Following muscle removal, soft tissue structures like ligament and joint capsule was retained. Then, the ends of the head and tail was smoothed. Four Kirschner wires were implanted along the longitudinal and transverse axes of L5 and L6 to assist to determine whether intervertebral activities occurred. Finally, the models were randomly grouped and stored at low temperature.

### Special internal fixation and fixture design and strain value measurement

Stress measurement of internal fixation of spine in vitro was rarely studied. Based on common internal fixation, special screws and connecting rods were designed and manufactured. They had the advantage of having a smooth platform, which could fit with strain gauges. According to the FE analysis results, the design position of the platform was set to the stress concentration area included the screw tail and the middle part of the connecting rod. For biomechanical experiments with high stress, fixture that could connect model and test instruments was also designed. The stable clamped model could also ensure the normal measurement of strain value.

Meanwhile, the special fixture could cooperate with the testing machine and torque wrench to produce different working conditions. The parameter of compressive preload and pure moment was consistent with the computer simulation of FE analysis. When the working condition reached the maximum, the strain value on the internal fixation would be measured. Static strain test analysis system (DH3821, Donghua test, Jiangsu, China), universal testing machine (SANS CMT4204, MTS System (CHINA) Co., Ltd., Shenzhen, China) and torque wrench (WEC2-030BN, WIZTANK, Eclatorq Technology Co., Ltd., Taiwan, China) were selected and used.

### Statistical analysis

SPSS 17.0 software (SPSS, Inc., Chicago, IL, USA) was performed for statistical analysis. Data were statistically analyzed based on the analysis of variance for repeated measures. Data were expressed as the mean ± standard deviation, and the normality of the data distribution was assessed using the Shapiro–Wilk test.

## Results

### Intact finite element model

Concerning the results of the intact L1–S1 vertebrae FE model, we took full advantage of Mimics 10.01, Geomagic Studio 12.0, Solidworks 2012, HyperMesh 13.0, and Patran/Nastran 2012 softwares. The high-quality FE model contained six vertebrae, five intervertebral discs and six ligaments; which also consisted of 368,233 tetrahedron elements and 79,722 nodes.

### Model validation

**Figure 4:**
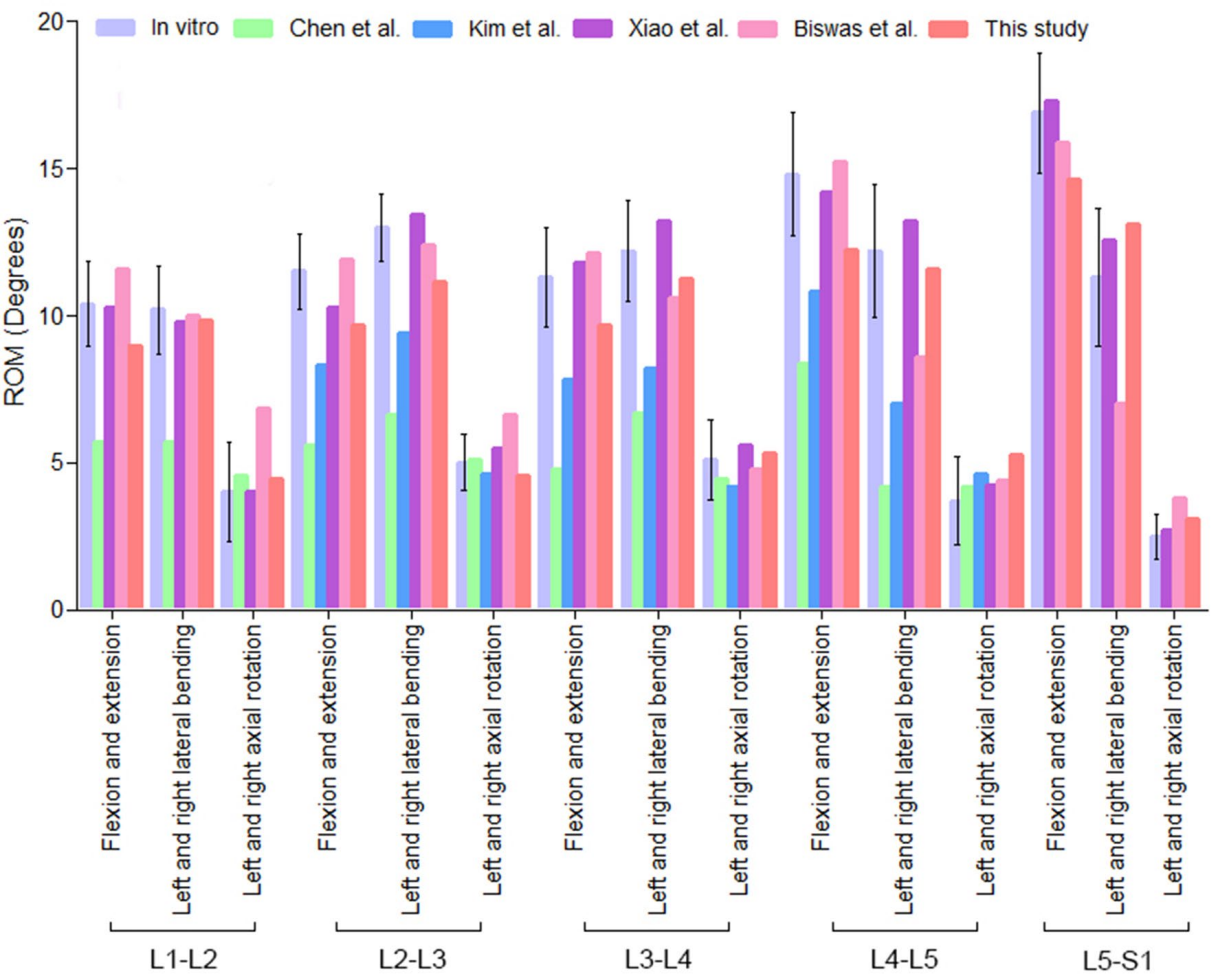
Comparison of ROM, between this study and the results reported by the previous literature.

Figure 4 indicated biomechanical test data in vitro and simulation results of four well-validated FE models in the literature were used for the comparison. The comparison of ROM between this intact FE model and previously published data under the combined flexion, extension, left–right lateral bending and left–right axial rotation modes were summarized. There were no obvious differences in the ROM between the intact FE model and the data published by the literature. Because all the data were conformed through normal human body parameters, the intact FE model could simulate the physiological movement of L1–S1 vertebrae. And its simulation performance was already to be used for further biomechanical studies by computer simulation.

### Three finite element models of posterior fixation of L4–L5 vertebrae

Based on the validated intact FE model, the L3–S1 model was selected to simulate posterior screw-rod system fixation surgery of L4–L5 vertebrae. The simplified pedicle screws and connecting rods were designed in Geomagic Studio 12.0 software and were assembled into different internal fixation systems on three same FE models. Here, the contact surfaces between screws and trajectories were defined as an infinite friction coefficient. Additionally, all components of the internal fixation system were recognized as a whole. Then, re-meshed models were established by using Hypermesh 13.0 software. With different points and orientations of pedicle screws, the Roy-Camille, Magerl, and Krag surgery FE models were ready for biomechanical studies.

**Figure 5:**
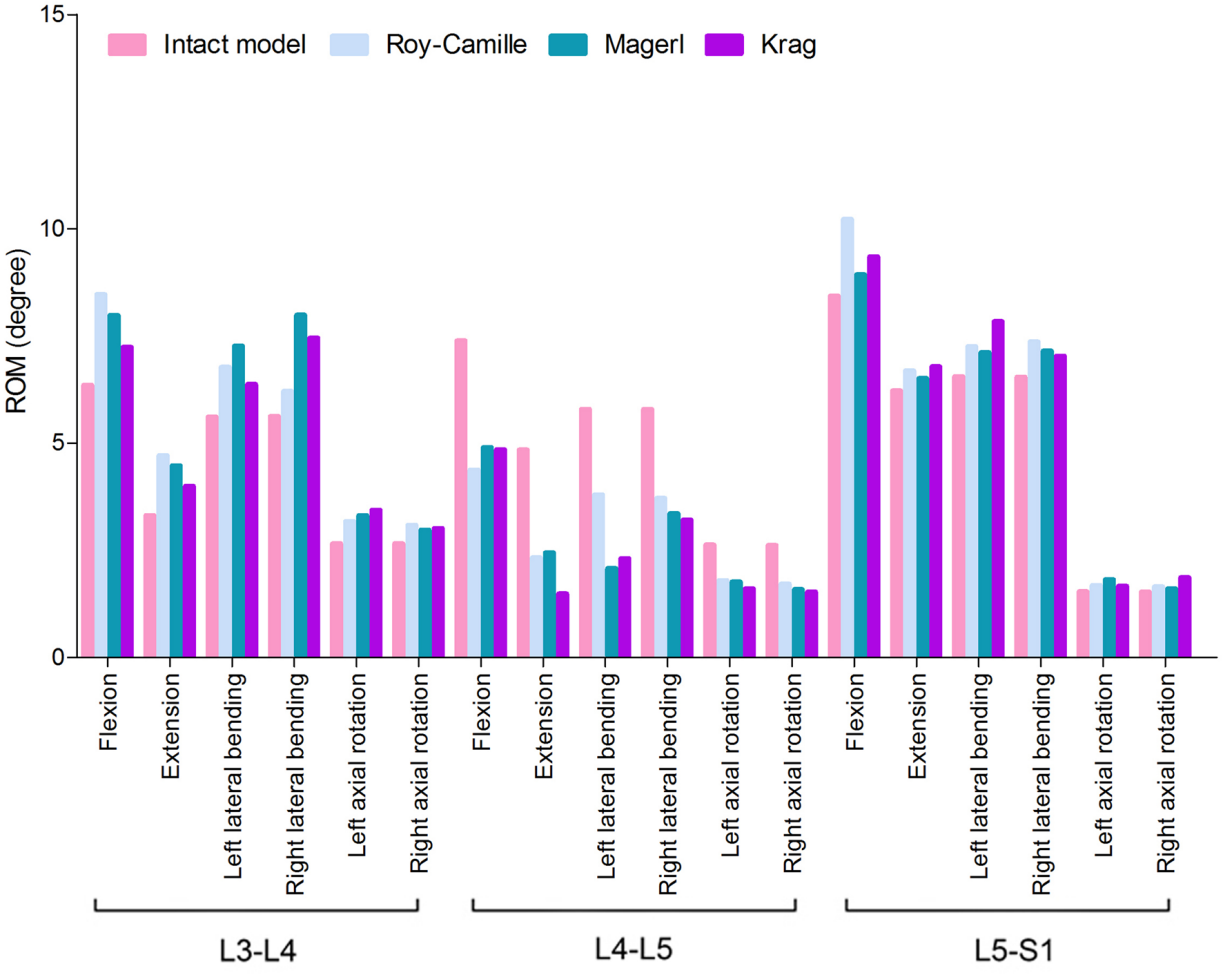
Comparison of ROM, between postoperative FE models and the intact FE model.

After the internal fixation of surgery, L4–L5 intervertebral ROMs of the different FE models were all reduced in comparison with the intact FE model (Fig. 5). It was worth noting that the changes of flexion and extension conditions were the most obvious. On the contrary, L3–L4 and L5–S1 intervertebral ROMs in different FE models slightly increased.

**Figure 6:**
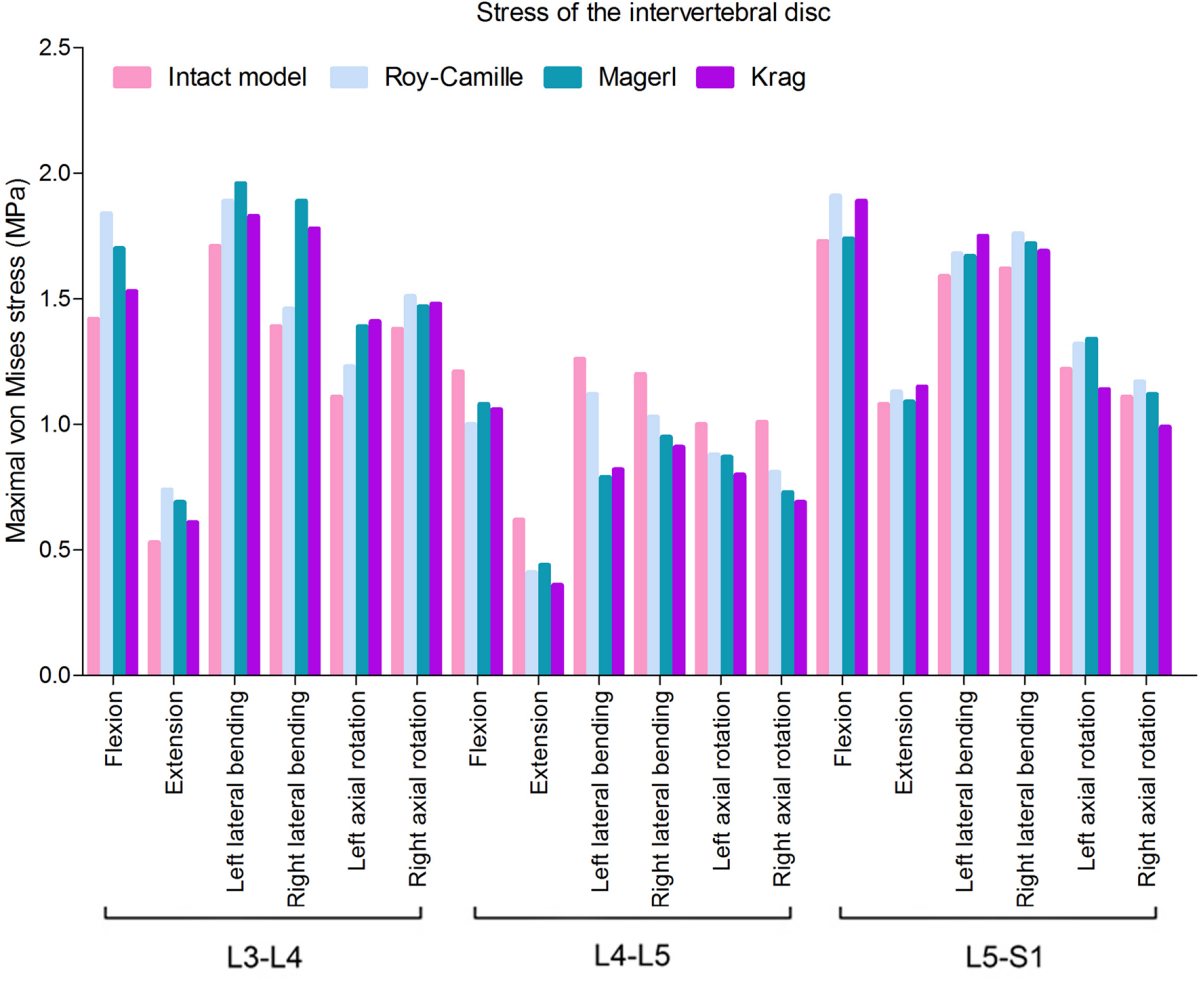
The von Mises stress of the adjacent-segment intervertebral discs in different FE models.

**Figure 7:**
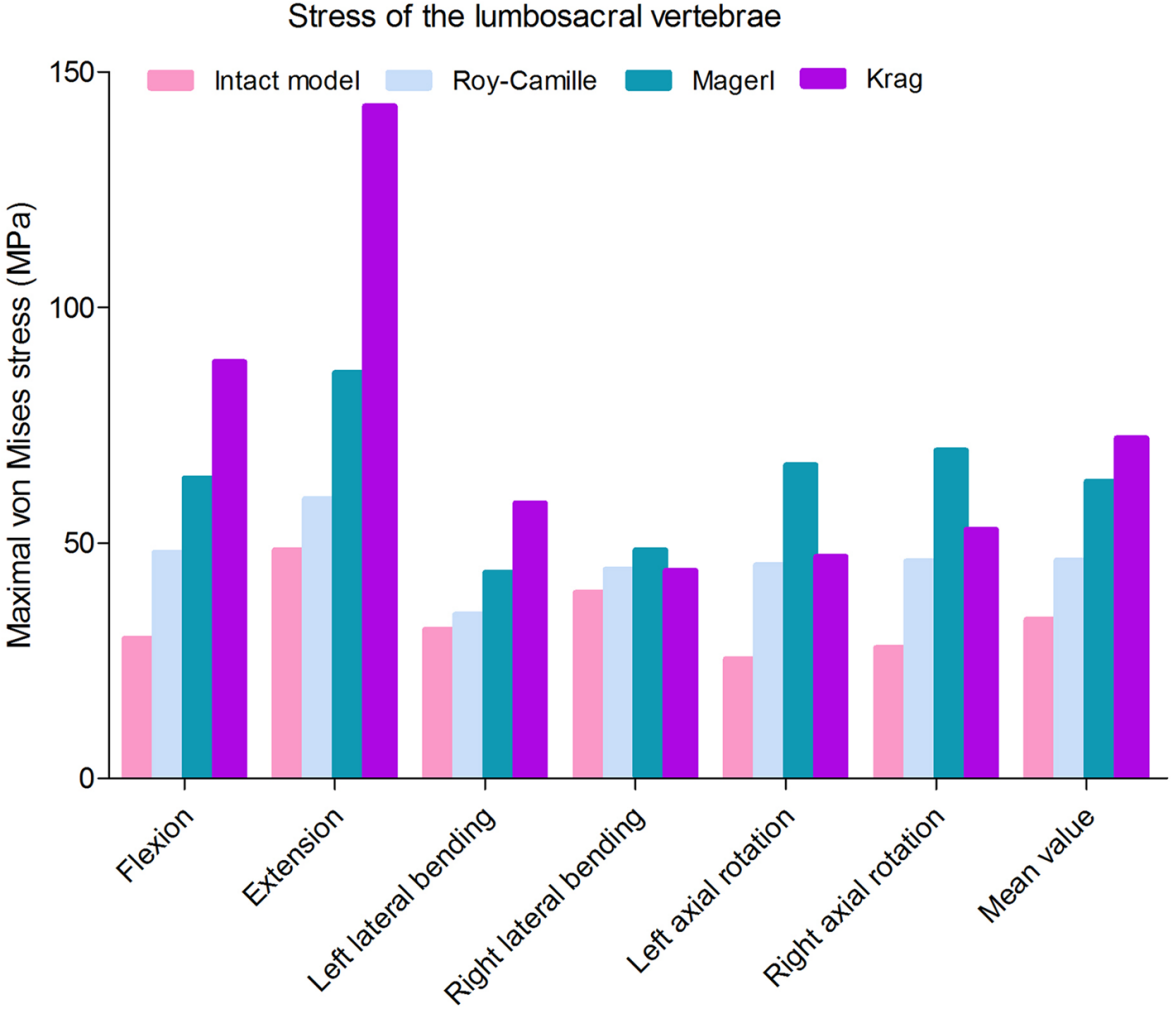
The von Mises stress of the lumbosacral vertebrae in different FE models.

**Figure 8:**
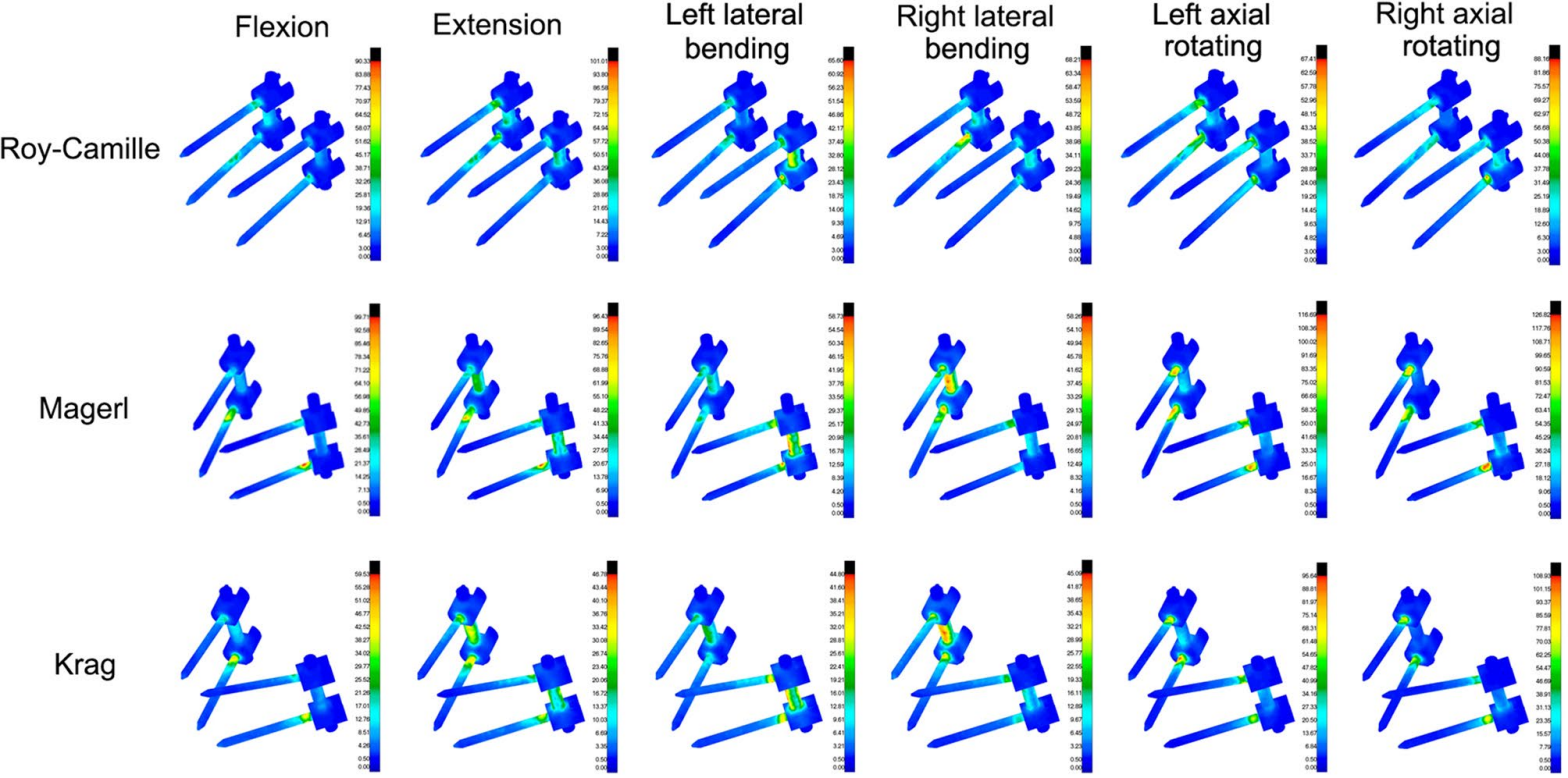
The stress distribution of screw-rod systems in three postoperative FE models under different conditions. According to the indicator diagram, red indicates the stress concentration area, while blue shows the stress dispersion area.

**Figure 9:**
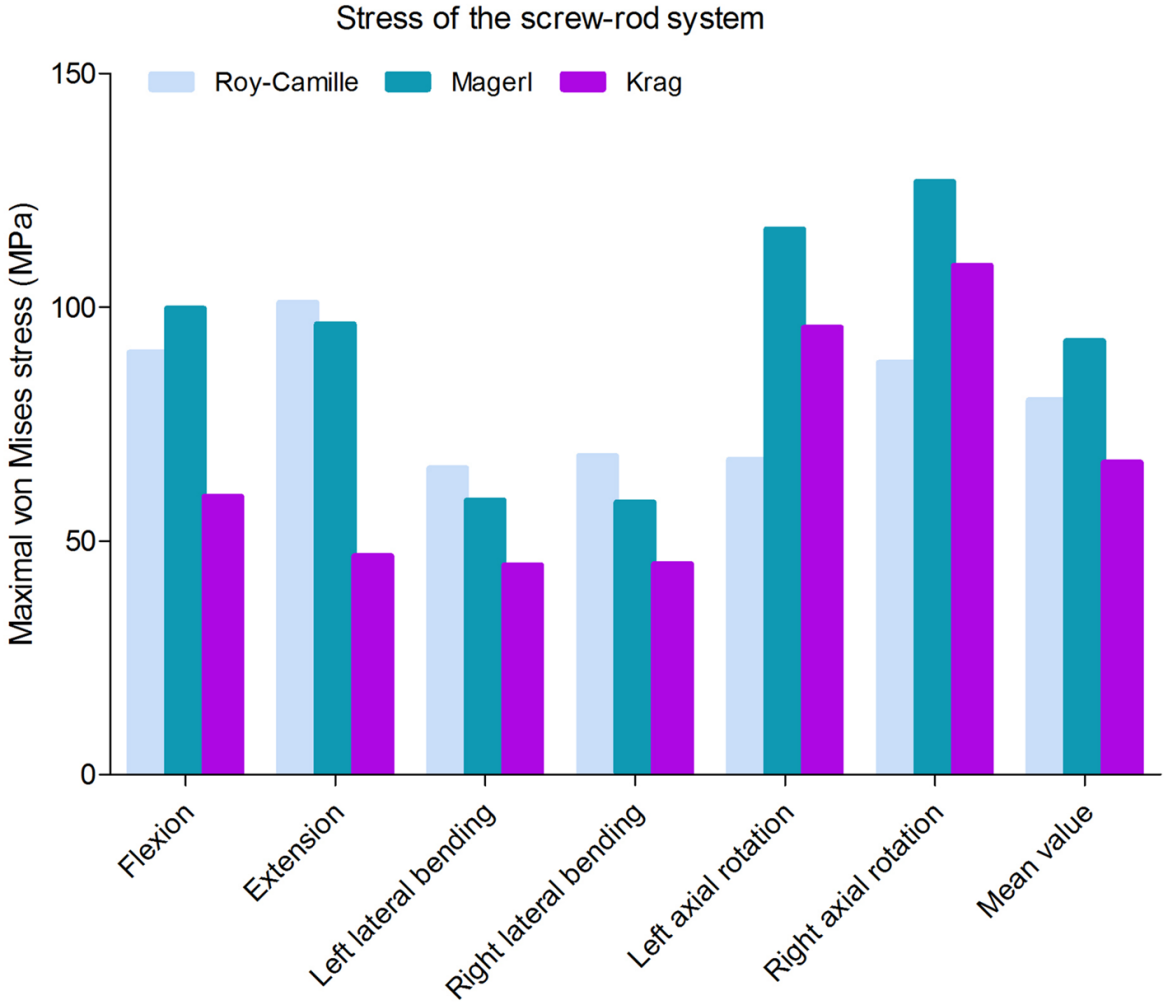
The von Mises stress of the internal fixation in three postoperative FE models under different conditions.

The Maximal von Mises stress of lumbosacral vertebrae, L3–L4 intervertebral disc, L4–L5 intervertebral disc and L5–S1 intervertebral disc in different postoperative models were different (Figs. 6 and 7). Greater stress concentrated areas were all observed on L3–L4 and L5–S1 intervertebral discs in these three postoperative FE models. Greater stress concentrated areas were observed on lumbosacral vertebrae in Krag FE model. For the internal fixation, the stress distribution of screw-rod system in three FE models was shown in Fig. 8. The Maximal von Mises stress areas concentrated on both the centre of connecting rods and roots of pedicle screws for all the models, especially during flexing, extension, left axial rotating and right axial rotating (Fig. 9). Interestingly, we noted that the stress distribution in Krag screw-rod system was more dispersive than others. The mean stress values of the three groups (Roy-Camille, Magerl, and Krag) were 80.12 MPa, 92.77 MPa and 66.80 MPa, respectively. Moreover, the maximum stress level for Roy-Camille and Magerl screw-rod systems were as much as 126.82 MPa and 101.01 MPa, respectively.

### Biomechanical test model preparation

**Figure 10:**
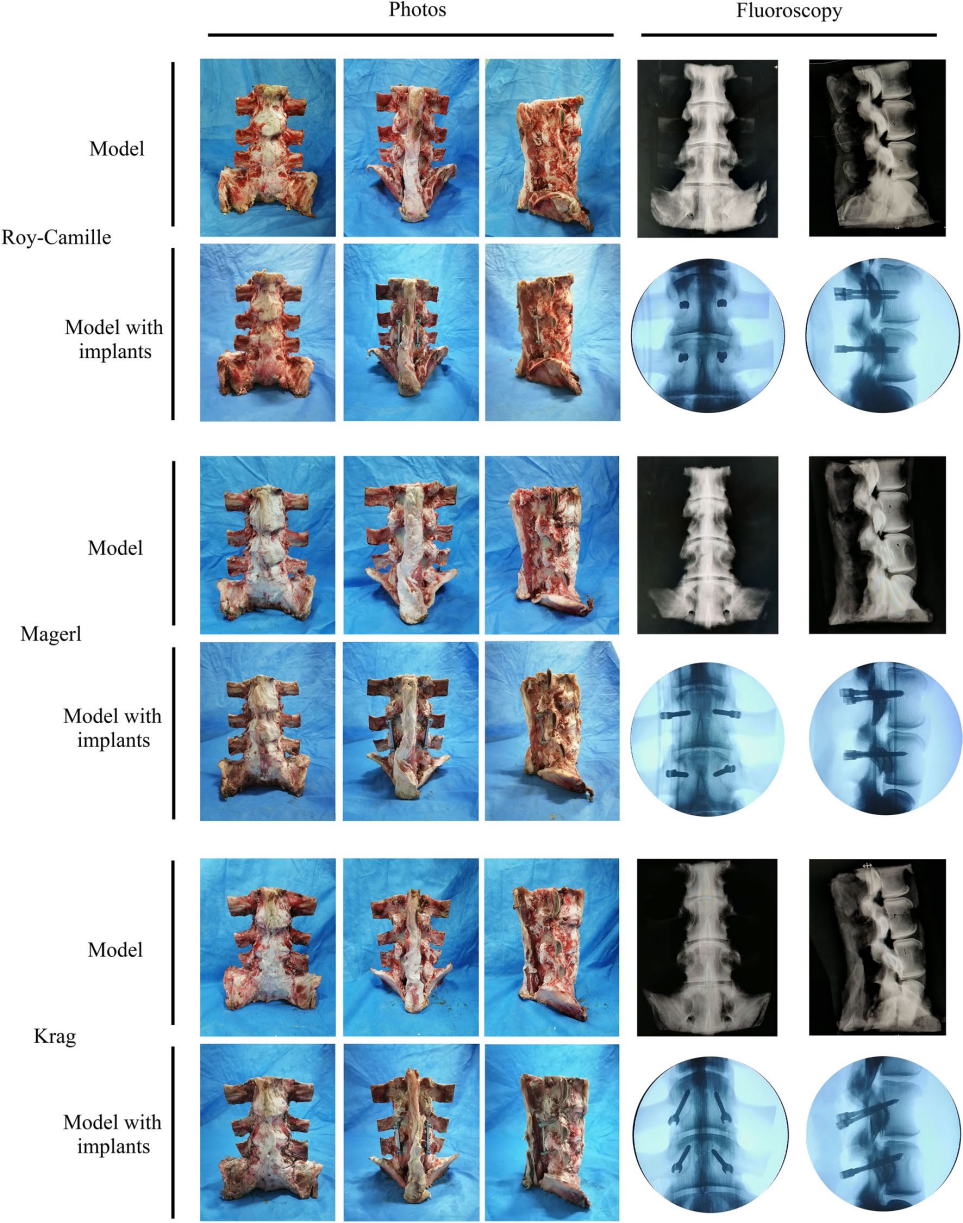
The processing of the models.

Following radiological examination, nine calf spines (L4–S2) were serially processed to removal muscle as well as keep soft tissue structures. Finally, intact vertebral bone, intervertebral discs, anterior longitudinal ligament, post longitudinal ligament, ligamentum flavum, interspinous ligament, supraspinous ligament, and articular capsule were retained. For the convenience of assembly, the ends of the head and tail was ground down by an angle grinder. Then, the models were grouped by the sortition randomization method. Low temperature storage of the models were reserved for further experiments. Then models would be assembly internal fixation following three different insertion techniques (Fig. 10).

### Special internal fixation and fixture design and strain value measurement

**Figure 11:**
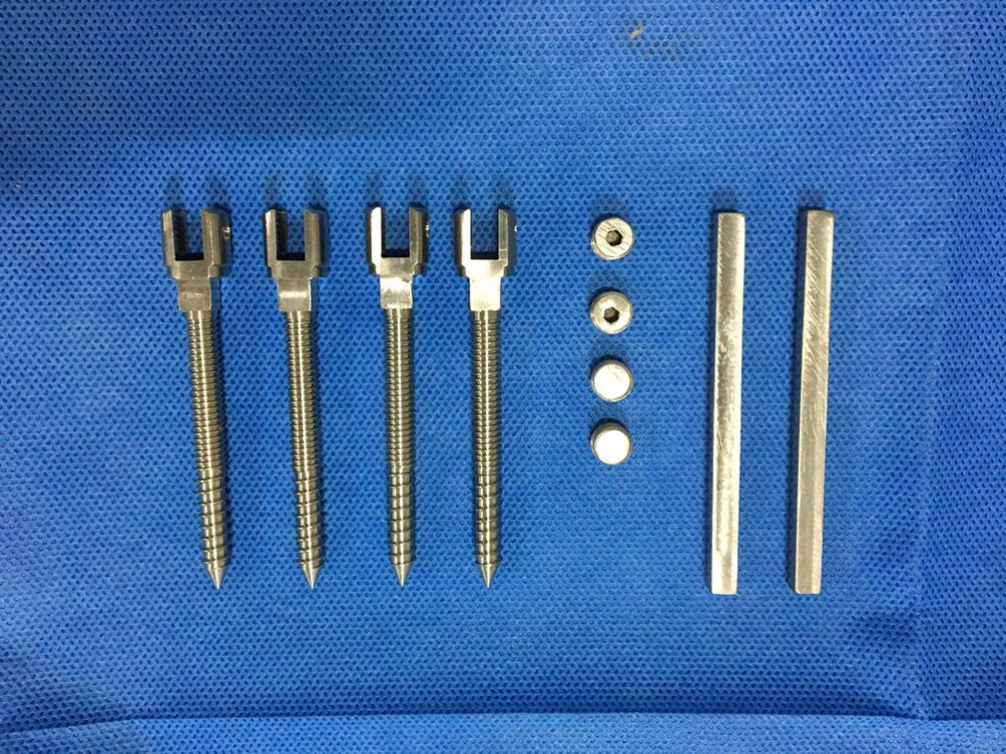
The special designed screws (diameter = 6.5 mm) and connecting rods (side length = 5.5 mm) for attaching strain gauges.

According to the results of the FE analysis, the stress concentration area was found. This provided a possibility for carrying out the measured test. Here, the special internal fixation were produced. Different from the previous internal fixation, special screws and connecting rods had a platform structure in the rear of screw and central region of rod (Fig. 11). These structures could be connected with strain gauges, which were used to capture the strain signal and transmit it to the static strain test analysis system.

**Figure 12:**
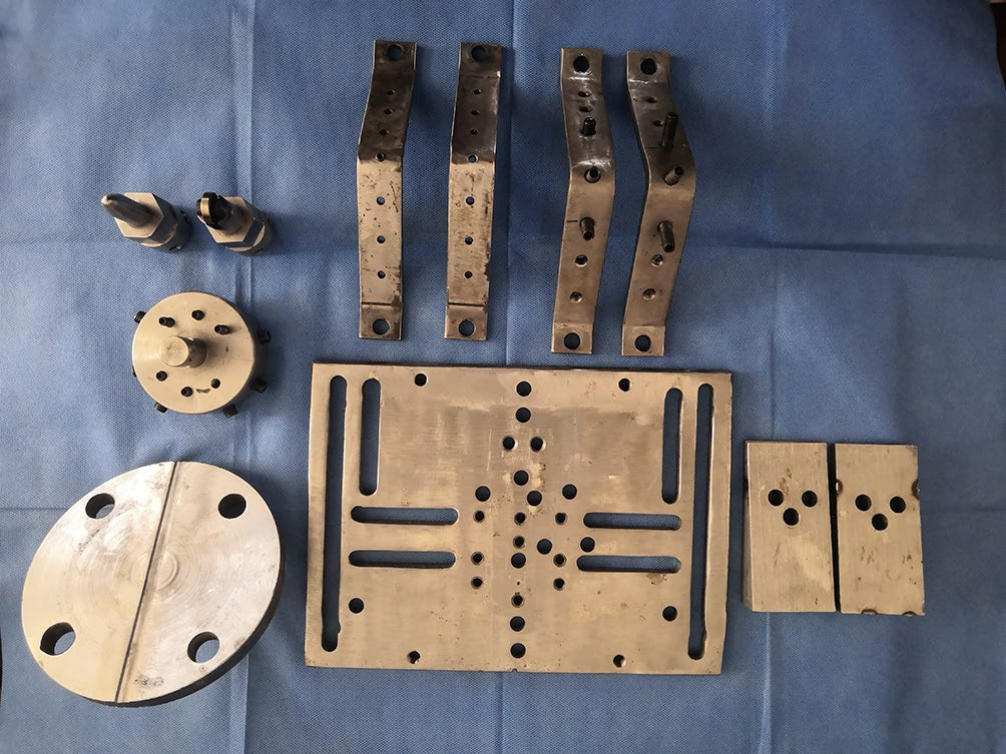
The special designed fixture for both fixing calf spine model and connecting with the testing machine.

On the other hand, the previous experiments were analyzed and evaluated comprehensively to assist to complete the design of special fixture. This study also brought a whole new tool (Fig. 12). Stable bottom components could provide different test angles of the model. The header component guaranteed the connection to the universal testing machine and drove models produce different working conditions.

**Figure 13:**
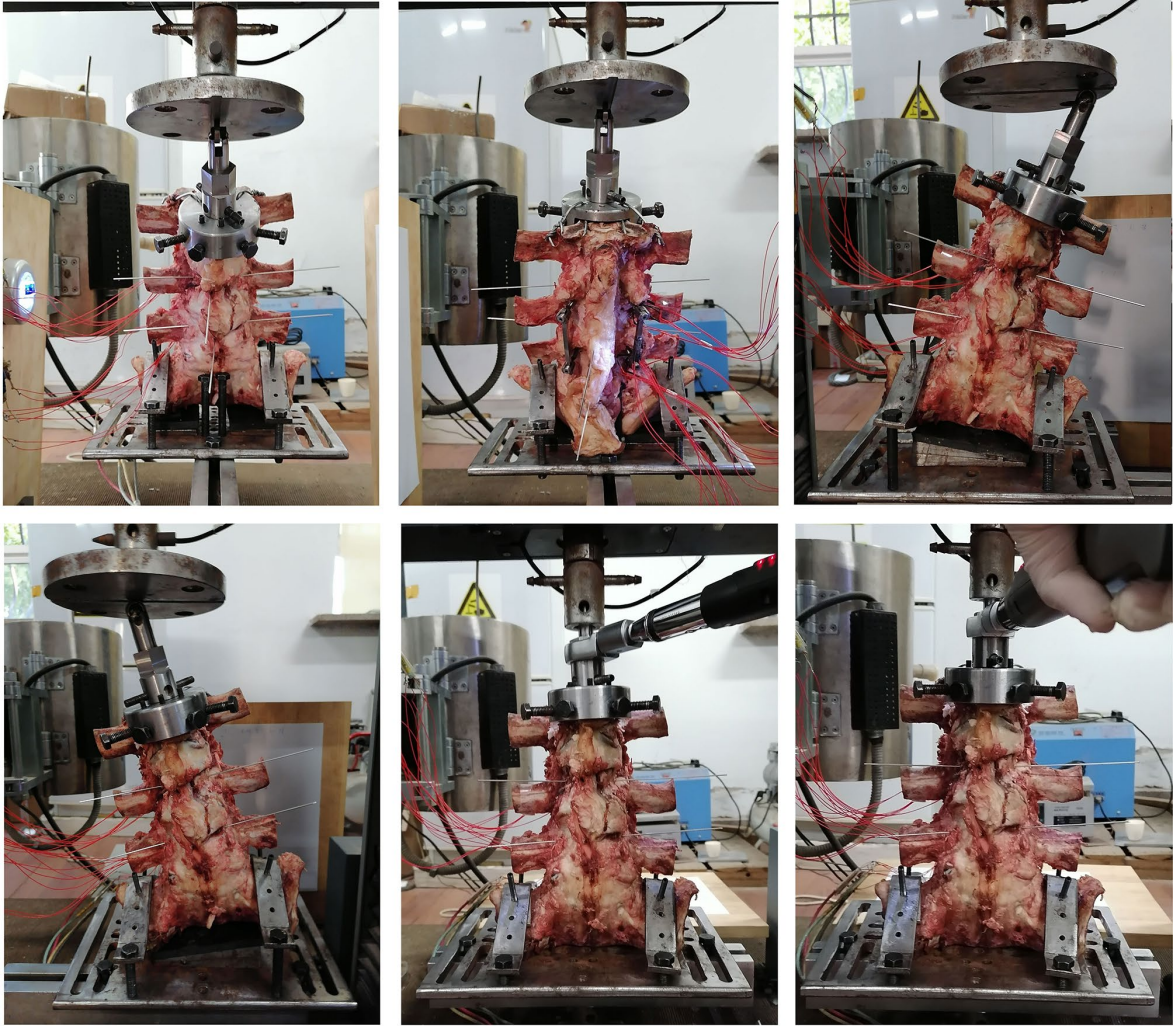
Simulation of six different working conditions on the test machine.

**Figure 14:**
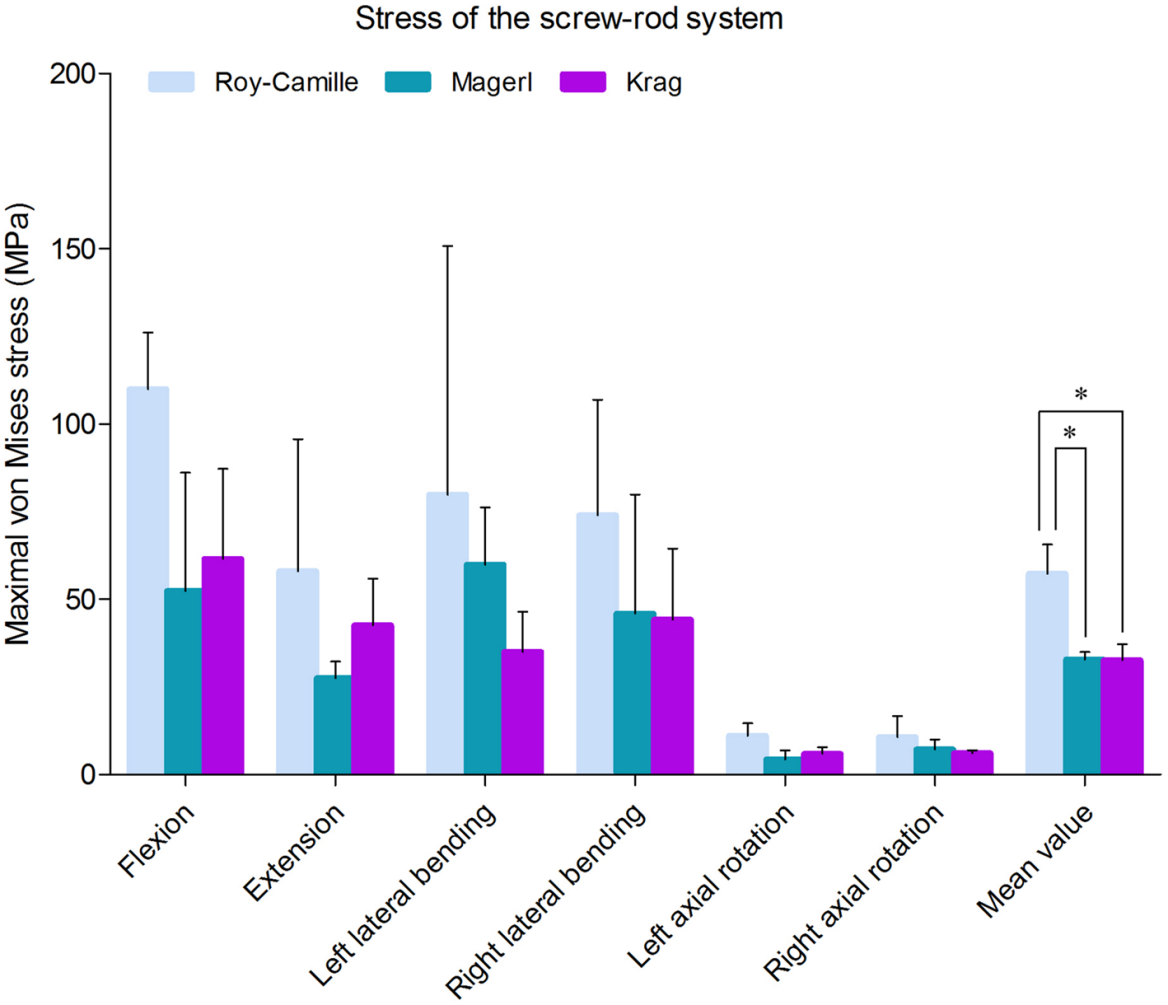
The stress value of screw-rod systems in three postoperative calf spine models under different conditions. (*: *P* < 0.05).

After internal fixation implantation and fixture assembly on the prepared calf spine, twelve strain gauges were attached to unilateral two screws and a connecting rod. Under the joint drive of fixture and testing machine, the model simulated different working conditions of normal physiological activities (Fig. 13). Intervertebral activities between L5 and L6 were found indirectly by judgement of the angle between kirschner wires. Static strain test analysis system captured the strain data at each point. Then, the stress value would be calculated by formula. Finally, stress extremes at each point were selected. From the results, under the same load conditions, flexion, extension and bilateral lateral bending were the working conditions in which the internal fixation stress was more concentrated. The mean stress values of Roy-Camille, Magerl, and Krag group were 57.30 MPa, 32.92 MPa and 32.58 MPa, respectively. Compared among three postoperative models, Roy-Camille insertion technique had the greatest impact on internal fixation and significantly more dangerous than the other two technologies with maximum value 156.35 MPa (Fig. 14).

The formulas for calculating stress is below.

$$\upsigma = {\text{E}} \times\upvarepsilon$$

(σ: stress, E: modulus elasticity , ε: strain).

## Discussion

With the increasing and aging trends of the population, low back pain has been the leading cause to disability^32^. The diagnosis and treatment of lower back pain have been the hot research fields that draw more and more attention. With the rapid development of radiological technology, the diagnosis of lower back pain becomes easy to be determined. Meanwhile, internal fixation techniques including implants and operation methods are being constantly improved. However, for those cases that undergo the spinal surgery, postoperative complications of fixation devices usually cause surgeons to worry.

The use of the pedicle screw has been more than half a century^2^. As the main force for multi-plane stability reconstruction of lumbar vertebra, pedicle screws are used in most of posterior lumbar surgery. There's no avoiding the fact that postoperative problems mainly including loosening and breakage become frequent and tricky. With more in-depth studies, pedicle screw loosening has been proved to closely related to the decrease of pullout strength and the change of insertion torque. The main influencing factors of pullout strength are density, insertion angle, insertion depth and reinsertion^33^. And bone density of vertebra is an important factor in pedicle screw instrumentation and contributes highest toward insertion torque (82%) and pull out strength (76%)^34^. Pull out strength increases with an increase in density and insertion depth. Whereas, insertion angle has no significant effect on both pull out strength and insertion torque. In the bilateral screw-rod system, reinsertion also has no significant effect on pullout strength^35^. A new research suggested that the roughness of surface is another important factor for insertion torque^36^. Incidence of screw breakage ranges between 2.6 and 60%^8–10^ and its occurrence represents a serious problem. A few of methods like the modified screw and dynamic stabilization system are created to cope with it. However, these attempts still are not able to completely remove the risk of breakage^12,37^. Meanwhile, cadaveric research and clinical retrospective study predict the uncertain results because of obvious limitations mainly including nonrepeatability, proof heterogenicity and large consumption. Therefore, there has been limited progress in biomechanical research. There are many frequently-used kinds of pedicle screw insertion techniques. The answer about relativity between insertion techniques and risk of breakage is not given throughout. Based on this, FE analysis combined with model measurement research can be regarded as a reliable approach for evaluating the biomechanical characteristics of different pedicle screw insertion techniques.

We drawn insights of FE analysis from previous literature and selected a healthy young volunteer to complete collection of imaging database. Mimics 10.01, Geomagic Studio 12.0, Solidworks 2012, Hypermesh 13.0, and Patran/Nastran 2012 softwares were performed to establish this intact FE model. To eliminate the difference of actual measurements and FE studies, five typical biomechanics data including in-vitro and FE studies were used for model validation^25,27–31^. ROM, commonly used in the step of comparison, was adopted by us. The values of flexion, extension, left–right lateral bending and left–right axial rotation movements of this intact FE model were in accordance with the numerical range of the included studies. Additionally, the mesh quantity with 368,233 tetrahedron elements and 79,722 nodes was moderate and acceptable. Therefore, our validated FE model could be used for further biomechanical researches and was different from previous reports. The reconstructed model was based on data from one healthy adult volunteer. This ensured the authenticity of the model. Combined with the processing advantages of different engineering software, the process of model establishment was optimized. This FE model had appropriate quantity of elements and nodes that reducing the computing burden will be beneficial to the study of stress distribution of the internal fixation system. Therefore, in this study, the accuracy of FE analysis results was guaranteed while the efficiency of model processing was improved.

Because of L4, L5 and S1 vertebra enduring the largest gravity in the lumbosacral vertebrae, L4–L5 intervertebral disc is often troubled by illness. Moreover, posterior lumbar surgery that mainly applied pedicle screws is widely used, now. Therefore, bilateral posterior pedicle screw-rod system fixation on L4 and L5 vertebra is the ideal surgery model for comparing different pedicle screw insertion techniques. Then, we extracted L3–S1 lumbosacral vertebrae from the intact FE model to perform this biomechanics study. By surgery simulation, mesh generation, materials properties definition, load applying and condition setting, three different models about pedicle screw insertion techniques following single-segment internal fixation were ready. The biomechanical characteristics of different models were compared. Previous studies have shown that lumbar interbody fixation are able to increase in ROM of adjacent segments of both sides^38^. Here, ROMs of L3–L4 and L5–S1 in three different postoperative models increased, compared to the intact model. The stiffness-increasing mechanism as the potential reason for the fixation-induced compensation is widely accepted. The stiffness-increasing effect protects the bridged segment from deformation, and transfers the load to the adjacent segments. This phenomenon can account for the higher kinematic and kinetic demand at the adjacent segments after implantation of internal fixation. Hsieh indicated that adjacent discs were subjected to the transferred loads from the instrumented segment^38^.

For the stress of internal fixation system, flexing, extension, left axial rotating and right axial rotating were the noteworthy working conditions that caused stress concentration. The difference of the pedicle screws’ points and orientations contributed mostly to the difference in stress distribution^39,40^. When the different points and orientations were selected, stress distribution of pedicle screws and rods would be changed consequently. After lumbar posterior surgery, screw-rod system became the center of stress. As we know, stress concentration might directly lead to breakage of the fixation system^19^. Because the pedicle screw belongs to the posterior internal fixation instrument, it is like the "crane" force structure that determines the stress concentration on the junction of thread and smooth. Screw breakage described by previous literatures often happen in this area^41^. This study result tied well with previous studies wherein the same was observed from all the three different postoperative models. Similarly, the rods connecting two pairs of pedicle screws up and down were under the major stress^39^. In the FE study section, the mean stress values of the three groups (Roy-Camille, Magerl, and Krag) were 80.12 MPa, 92.77 MPa and 66.80 MPa, respectively. Krag with the minimum mean stress value seemed better than the other two insertion methods. To verify the results of FE analysis, biomechanical test with nine calf spines were performed. According to the characteristics of concentrated stress areas, special internal fixation and fixture were designed to well simulate six working conditions. Choosing the same load conditions as the FE study, the strain value was obtained and calculated into stress value. Finally, results of biomechanical test showed that the mean stress values of Roy-Camille, Magerl, and Krag group were 57.30 MPa, 32.92 MPa and 32.58 MPa, respectively. It was not difficult to see that Krag and Magerl were two more secure insertion method for protecting the posterior pedicle screw-rod system. Additionally, flexing, extension, left lateral bending and right lateral bending easily caused stress concentration. Taken together FE analysis and biomechanical test results, Krag insertion method was better than the other two with a lower risk of breakage. Krag insertion technique emphasizes on the trajectory that stands in sharp contrast to other insertion techniques. Screw-rod system based on Krag insertion technique transfers the stress to the lower part. This allows stress to be dispersed in the lumbar spine with internal fixation. In contrast, screws implanted by Roy-Camille and Magerl insertion technique parallel to the upper and lower endplates in the sagittal position. At that time, the tail of the screw is at right angles to the connecting rod. This may be the cause of relatively high stress of screw-rod system. Flexion and extension were still the most dangerous conditions after lumbar posterior surgery. Although the data was not sufficient, lateral bending and rotation could not be underestimated, too. Processed by software, FE model was made as idealized as possible, but biological simulations of tiny structures were difficult to complete, such as the motion of the joint capsule. The inconsistencies in the two experiments were related to the nuances of different models.

It should be noted that the comprehensive analysis method adopted in this study was an innovation. As we known, FE analysis is widely used to complete mechanical analysis of internal fixation and bone. High emulation, low cost and time saving are its great advantages. However, biomechanical test is in stark contrast to the former. Its advantage is that repeated tests can be carried out with multiple specimens. Strain gauge is a common method of stress measurement and often used for mechanical and material testing. But, in the previous researches, combination with FE analysis and strain gauge measurement is rare. Oral biomechanics was the first to adopt this approach. Palamara et al. investigated the variations in strains in enamel under different patterns of occlusal loading. Strains predicted from the FE model were in excellent agreement with the strain gauge measurements^42^. A few of studies were performed by the similar method and continuously improved its reliability^43–45^. In recent years, this method began to be transplanted into orthopedic stress research. Bone surface strains of the radius and ulna in mouse has been studied by Begonia et al. via using the similar method^46^. Then, Gao et al. believed this combination method was a feasible way and showed the strain distribution of axial compressive load of rat tibia^47^. These satisfactory results proved the validity again. In this study, we adopted this research method, but the object of study was no longer human tissues, but internal fixations with metallic material properties. Strain gauge placement is a difficult point in this kind of measurement. Therefore, special screws and connecting rods with smooth platform was designed. Then, it is possible to measure the strain of internal fixations. In this study, the results of FE analysis were mostly in agreement with the measured results of strain gauge.

The final results of this combination test will make some difference in the clinical choice of pedicle screw insertion technique. Based on the Krag technique, the much safer insertion techniques will be designed and promoted. Additionally, developing decision support system based on this study for pedicle screw fixation can supply the personalized therapy and precision medicine for patients suffering from lumbar disease.

There were still some limitations in the present studies. Limited to the tremendous computing workload, elements and nodes of the intact model were moderate rather than elaborate. Based on data from different individuals, multiple FE model of lumbar spine would help to verify the results of FE studies. This might be a better representation compared to the combination of FE analysis and biomechanical test. Current pedicle screw insertion techniques are diverse. The differences are mainly in entry point and insertion orientations. Only three common and typical pedicle screw insertion techniques were selected for the comparison. This limitation of selection had impact on the research comprehensiveness. Thus, the results of other techniques were unknown and needed further studies. In the biomechanical test section, the sample size was still insufficient. There were differences from selected calf vertebra and human vertebra in the number, shape and size. The simulation of the working condition should be more ideal, more close to the FE study, more in line with the physiological state. There were some differences in the stress values measured from the FE analysis and the biomechanical test, which was related to the experimental environment, the model difference, experimental measurement and calculation method. The most authentic and reliable results could be attained by combining with large sample clinical data. Additionally, more efforts should be done to explore the influences caused by screw size, pedicle fill, bone density, and bone structures.

In summary, compared with others, Krag insertion technique could reduce the stress concentration of screw-rod system. Without the consideration of other factors, Krag insertion technique was safer for reconstructing the stability of lumbar vertebra, due to its potentially low risk of fixation breakage. Additonally, combination of FE analysis and strain gauge test was a good selection for biomechanical study of spinal internal fixation.
